# Comparison of astigmatism prediction error taken with the Pentacam measurements, Baylor nomogram, and Barrett formula for toric intraocular lens implantation

**DOI:** 10.1186/s12886-017-0550-z

**Published:** 2017-08-24

**Authors:** Dae-Young Park, Dong Hui Lim, Sungsoon Hwang, Joo Hyun, Tae-Young Chung

**Affiliations:** 1 Department of Ophthalmology, Samsung Medical Center, Sungkyunkwan University School of Medicine, #81 Irwon-ro, Gangnam-gu, Seoul, 06351 South Korea; 2 Department of Preventive Medicine, Graduate School, The Catholic University of Korea, Seoul, South Korea; 3 Department of Ophthalmology, Saevit Eye Hospital, Goyang, South Korea

**Keywords:** Astigmatism, Toric intraocular lens, Corneal posterior astigmatism, Pentacam, Astigmatism prediction error, Vector, IOLMaster, Baylor nomogram, Barrett formula

## Abstract

**Background:**

To evaluate and compare the astigmatism prediction errors taken with the Pentacam measurements, Baylor nomogram, and Barrett formula for toric intraocular lens (IOL) implantation.

**Methods:**

Phacoemulsification with toric Precizon IOL implantation was performed in 41 eyes with corneal astigmatism (range, 1 to 5 diopters (D)) determined by IOLMaster and SimK on Pentacam. Preoperative corneal astigmatism measurements were obtained from IOLMaster readings (IOLMaster, Baylor-IOLMaster, and Barrett-IOLMaster) and Pentacam readings (SimK, Baylor-SimK, Barrett-SimK, wavefront, true net power, total corneal refractive power, and vector derived by manual vector summation using corneal front and back astigmatism). Prediction error and intraclass correlation coefficient (ICC) between the measured (or calculated) astigmatism by IOLMaster and Pentacam and the estimated corneal astigmatism estimated by IOL toricity power and residual astigmatism were determined.

**Results:**

The centroid errors in prediction error with IOLMaster, SimK, Baylor-IOLMaster, Baylor-SimK, Barrett-IOLMaster, Barrett-SimK, wavefront, true net power, total corneal refractive power, and vector were 0.59@103, 0.61 @103, 0.37@161, 0.41@162, 0.24@171, 0.36@162, 0.42@106, 0.04@8, 0.07@82, and 0.03@82, respectively, in with-the-rule (WTR) astigmatism eyes at postoperative 3-month. They were 0.22@87, 0.20@74, 0.16@21, 0.54@10, 0.43@3, 0.33@19, 0.51@25, 0.31@58, 0.29@50, and 0.14@50 in against-the-rule (ATR) astigmatism eyes. Of the ten modalities, vector showed the lowest WTR astigmatism prediction error and the highest ICC between the predicted and the estimated corneal astigmatism for both WTR and ATR eyes.

**Conclusion:**

Vector summation using anterior and posterior corneal surface power taken with the Pentacam yields the least astigmatism prediction error and is a promising tool for determining toric IOL cylinder power.

## Background

Delicate-tuned refractive outcome is essential to all patients with pre-existing corneal astigmatism for toric intraocular lens (IOL) implantation. Several factors can contribute to residual refractive astigmatism error, including surgical induced corneal astigmatism (SICA), errors in toric IOL alignment, and methodologic error in predicting the toricity of IOL power [1]. Notably, most calculations for corneal astigmatism are based on anterior keratometry which can cause predictable error in both with-the-rule (WTR) and against-the-rule (ATR) astigmatism presumably due to the neglect of posterior astigmatism. A previous study has demostrated that posterior corneal astigmatism must not be neglected in predicting residual refractive astigmatism in toric intraocular lens implantation, because posterior corneal surface has significant influence on total corneal astigmatism [2, 3].

Unfortunately, current keratometry or corneal topography does not appear to reflect the true corneal astigmatism perfectly. To overcome this disadvantage, several solutions have been proposed. The Baylor nomogram and Barret toric calculation considering predicting values of posterior corneal astigmatism have been suggested [4, 5]. The new Schiempflug imaging devices including Pentacam and Galilei can allow us to obtain the keratometry (K) value of posterior corneal surface and calculate the true power of corneal astigmatism by combining the anterior corneal surface power using the net summation or ray tracing assay. With the Galilei system, Koch et al. have demonstrated that its ATR was successfully corrected, while there are still rooms for improvements for WTR [4]. Nevertheless, these vigorous attempts to predict accurate corneal astigmatism do not always guarantee a fine refractive outcome for toric IOL implantation. Therefore, the objective of this study was to evaluate and compare the astigmatism prediction errors taken with the Pentacam measurements, Baylor nomogram, and Barrett formula for toric IOL implantation.

## Methods

### Patients

Consecutive medical records of patients who had undergone cataract extraction with toric IOL implantation by one surgeon (T-Y.C.) between Aug 2015 and Feb 2016 at Samsung medical center, Seoul, South Korea, were retrospectively reviewed. Approval was obtained from the Institutional Review Board of Samsung Medical Hospital. Inclusion criteria were:

(1) qualified scans with IOLMaster (software version 5.02, Carl Zeiss Meditec AG, Jena, Germany) and Pentacam HR (software version 1.17r24, Oculus, Wetzlar, Germany), (2) no previous ocular trauma or surgery, (3) no ocular surface disease, (4) Precizon toric IOL (models 56501TY to 56514TY, Ophtec BV, Nethelands). Eyes were divided into two groups depending on the anterior corneal steep meridian measured by IOLMaster as follows: (1) WTR eyes with corneal steep meridian at 60 to 120 degrees, and (2) ATR eyes with corneal steep meridian at 0 to 30 degrees or 150 to 180 degrees. Patients with oblique corneal astigmatism (steep corneal meridian at 30 to 60 degrees or 120 to 150 degrees) were excluded from analysis.

### Surgical procedure

To avoid ocular torsional misalignments, the meridian of the incision was marked for all patients seating upright at slit-lamp using horizontal slit beam before surgery. One experienced surgeon (T-Y.C.) performed all surgeries under topical anesthesia. Phacoemulsification was performed with a 2.75 mm temporal clear corneal incision and a 3-plane clear corneal incision. Two small sideport incisions were created. They were located at approximately 30 and 150 degrees. Bimanual cortex removal was performed. The spherical equivalent power was determined using the results from SRK/T and Haigis formulas based on the keratometry and axial length of the IOLMaster. The selection of the IOL cylinder was based on the calculated astigmatism, vector, using the Precizion toric IOL calculator (http://calculator.ophtec.com/calculator-choice, accessed October 1, 2015). The SICA of the operating surgeon was assumed as 0.50 D for all cases. Vector was determined as an integration of anterior and posterior corneal surface astigmatism values (3 mm zone, keratometry index = 1.376 and 1.336, respectively) measured by Pentacam using vector summation based on Alpins method of vector analysis [6].

### Corneal astigmatism measurements and calculations

Keratometry measurement were measured preoperatively from IOLMaster (reflecting the surface of cornea with diameter of 2.5 mm) and Pentacam. Preoperative anterior chamber depth (ACD) was obtained from Pentacam. The astigmatism values of of Pentacam were evaluated in five different ways: simulated keratometry (SimK, on the cataract preoperative display, keratometriy index = 1.3375), wavefront (4.0 mm apex/zone setting on the cataract preoperative display, Zernike analysis and corneal wavefront calculated via ray tracing), 4-mm-zone true net power (TNP, 4.0 mm apex/ring setting on the power distribution map, based on a Gaussian optics formula), 4-mm-zone total corneal refractive power (TCP, 4.0 mm apex/ring setting, on the power distribution map, calculated by ray tracing using the Snell law, taking account of corneal thickness), and vector. Lastly, the Baylor toric nomograms [4] and Barrett toric calculators (http://www.ascrs.org/barrett-toric- calculator, v1.05, accessed April 1, 2016) [7] were utilized to determine the four types of astigmatism values using the IOLMaster keratometry values or SimK: Baylor-IOLMaster (application of the Baylor nomogram using a keratometry of IOLMaster), Baylor-SimK (application of the Baylor nomogram using SimK), Barrett-IOLMaster (taken from the Barrett toric calculator using a keratometry of IOLMaster), Barrett-SimK (taken from the Barrett toric calculator using SimK). Collectively, ten modalities of measured (or calculated) corneal astigmatism were determined. All measurements were performed by the same experienced examiner. Quality of keratometry readings of IOLMaster can be verified by visually checking the quality of the readings. Schiempflug camera examinations reaching good quality (image quality as ‘OK’) were taken. The corneal curvature measurements by both IOLMaster and Pentacam have been proven to be repeatable [8].

### Aggregate in surgical induced astigmatism change and residual astigmatism

Anterior and posterior corneal surface powers from Pentacam were measured at postoperative 1- and 3- month. An individual surgical induced astigmatism change (SICA) per case was obtained by using a vector summation of the pre- and post- operative values of vector from Pentacam. We aggregated SICA and postoperative manifest cylinder error and presented them as astigmatism centroid [9].

### Accuracy analysis for astigmatism measurements

To determine the accuracy of astigmatism measurements, we analyzed the prediction error and intraclass correlation coefficient (ICC) between the estimated corneal astigmatism and the measured (or calcualted) corneal astigmatism obtained by the ten modalities. To evaluate the prediction error, we firstly estimated the estimated preoperative corneal astigmatism. To avoid noise associated with incorrect IOL alignment for estimation of actual preoperative corneal astigmatism, we accessed an actual toric IOL axis alignment from anterior segment photography at postoperative 1- and 3- month rather than intented axis. We then calculated the estimated preoperative corneal astigmatism (actual preoperativel corneal astigmatism) based on the following eq.:

$$ \mathbf{Postoperative}\mathbf{manifest}\mathbf{cylinder}\mathbf{error}=\mathbf{Estimated}\mathbf{preoperative}\mathbf{corneal}\mathbf{astigmatism}+\mathbf{Toric}\ \mathbf{IOL}\ \mathbf{cylinder}\ \left(\mathbf{steepest}\mathbf{meridian}\right)+\mathbf{SICA} $$

We then calculated the prediction error using the following eq. [4]:

$$ \mathbf{Prediction}\mathbf{error}=\mathbf{Measured}\ \left(\mathbf{or}\mathbf{calculated}\right)\ \mathbf{preoperative}\mathbf{corneal}\mathbf{astigmatism}\ \mathbf{by}\ \mathbf{each}\mathbf{modality}-\mathbf{Estimated}\mathbf{preoperative}\mathbf{corneal}\mathbf{astigmatism} $$

To report the aggregate results of prediction error of each modality in a clinically meaningful way, the centroid error in prediction error was calculated and displayed on double-angle plots as described previously [4]. In a subset analysis, the prediction error was separately analyzed along with the horizontal and vertical axis in the double-angle plot of the predictions error as WTR/ATR and oblique prediction error, respectively [4]. Negative value in WTR/ATR prediction error could be interpreted as overcorrection in WTR astigmatism and undercorrection in ATR astigmatism, respectively. The oblique prediction error indicated the oblique astigmatism along 135 degrees (positive value) and 45 degrees (negative value). All valuables were corrected to the corneal plane using SRK/T with a refractive vergence formula [10].

ICC representing precision or reliability [11] was used to investigate the degree of resemblance between the measured (or calcualted) preoperative corneal astigmatism and the estimated preoperative corneal astigmatism. Similar to the way of prediction error analysis as described above, ICC was also separately analyzed for WTR/ATR and oblique ICC. ICC of more than 0.8 indicates an excellent agreement [11].

### Statistics

To detect a prediction error by 0.2 D, sample size was calculated. The standard deviation of prediction error in a preliminary study was shown as approximately 0.5 D. By input of a test power of 80% and a significant level of 5%, 41 eyes were determined. A normality test was conducted (Shapiro-Wilk test). Significant results were subsequently evaluated using a paired t test or Wilcoxon signed-rank test. Bonferroni correction was used for multiple comparisons. Statistical analysis was performed using SAS version 9.4 (SAS Institute, Cary, NC) and R 3.2.5 (Vienna, Austria; http://www.R-project.org/). A *P* value of less than 0.05 was considered as statistically significant.

## Results

### Baseline characteristics

Forty-one eyes of 34 patients (15 men and 19 women) were enrolled in this study. The mean age of the 34 patients was 61.3 ± 10.6 (SD, standard deviation) years (range, 41 to 83 years). Their mean spherical IOL power was 19.4 ± 3.8 D (diopters) (range, 15.5 to 25.5 D). Their mean ACD was 2.80 ± 0.42 mm (range, 1.91 to 3.42 mm). Their mean axial length measured by IOLMaster was 23.98 ± 0.97 mm (range, 22.01 to 25.57 mm). Their preoperative measured (or calcualted) corneal astigmatism by the ten modalities are summarized in Table 1. The posterior corneal astigmatism value measured by Pentacam was 0.56 ± 0.21 D (range, 0.20 to 1.0 D) in WTR eye and 0.26 ± 0.20 D (range, 0.10 to 0.70 D) in ATR eyes. The centroid mean values of postoperative 1- and 3- month manifest cylinder error were 0.02 @ 0 and 0.15 @ 21, respectively, in WTR eyes and 0.19 @ 164 and 0.27 @ 170, respectively, in ATR eyes. The centroid mean values (with absolute mean) of **SICA at postoperative 1- and 3- month were 0.25 @ 89 (0.44 ± 0.20 D) and 0.24 @ 81 (0.51 ± 0.28 D), respectively, in WTR eyes, and 0.65 @ 94 (0.74 ± 0.60 D) and 0.45 @ 81 (0.68 ± 0.27 D), respectively, in ATR eyes.**

**Table 1:** Preoperative measured (or calculated) corneal astigmatism measurements (D) by each modality

Modality	WTR eyes (*n* = 24)	ATR eyes (*n* = 17)
	Mean ± SD (range)	
IOLMaster	2.25 ± 1.04 (1.00–4.54)	1.39 ± 0.56 (1.00–2.19)
SimK	2.26 ± 1.02 (1.10–4.30)	1.44 ± 0.32 (1.10–2.10)
Baylor - IOLMaster	1.43 ± 0.92 (0.50–3.57)	1.78 ± 0.80 (0.50–2.50)
Baylor - SimK	1.37 ± 0.91 (0.50–3.02)	1.98 ± 0.30 (1.50–2.50)
Barrett - IOLMaster	1.59 ± 0.84 (0.50–3.49)	1.96 ± 0.54 (1.16–3.04)
Barrett - SimK	1.52 ± 0.76 (0.55–3.27)	1.74 ± 0.38 (1.19–2.39)
Wavefront	2.06 ± 1.12 (0.50–4.10)	1.88 ± 0.40 (1.00–2.50)
TNP	1.99 ± 0.98 (0.60–4.20)	1.44 ± 0.50 (0.30–2.10)
TCP	2.11 ± 1.07 (0.60–4.50)	1.54 ± 0.53 (0.30–2.20)
Vector	1.71 ± 0.93 (0.53–3.32)	1.49 ± 0.33 (0.92–2.09)

*D* diopters, *WTR* with-the-rule, *ATR* against-the-rule, *TNP* true net power, *TCP* total corneal refractive power

### Corneal astigmatism prediction errors

**Fig. 1:**
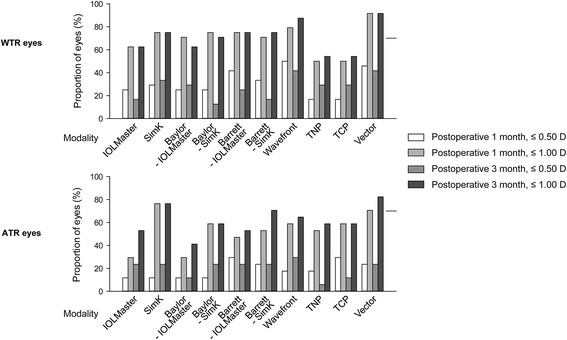
Proportion of eyes with absolute prediction errors within ±0.50 D and ±1.00 D by modality. WTR = with-the-rule, ATR = against-the-rule, TNP = true net power, TCP = total corneal refractive power

**Fig. 2:**
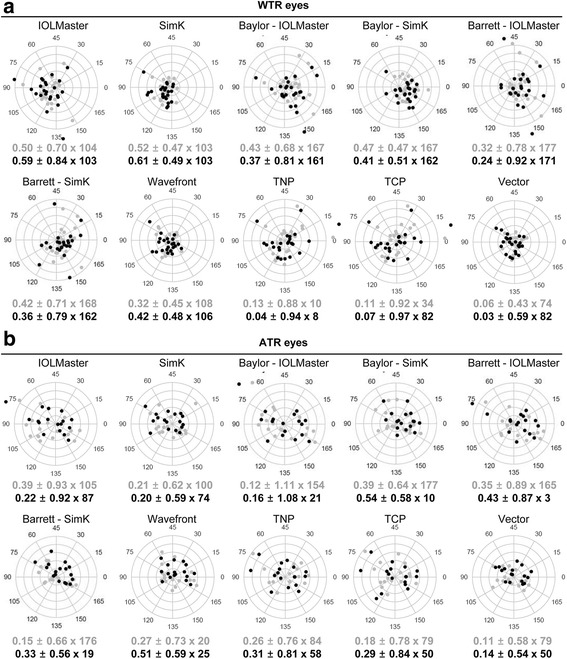
Double-angle plots of prediction errors in WTR (**a**) and ATR (**b**) eyes. Centroid errors in prediction errors at posteoperative 1- (gray color) and 3-month (black color) were shown. Each ring = 0.5 D (diopters). WTR = with-the-rule, ATR = against-the-rule, TNP = true net power, TCP = total corneal refractive power

The proportion of eyes with an absolute prediction error within ±0.50 D and ±1.00 D according to each modality was shown in Fig. 1. The higher proportion over 70% at both postoperative 1- and 3- month was observed in SimK, Barrett-IOLMatser, Barrett-SimK, wavefront, and vector of WTR eyes, and in SimK and vector of ATR eyes. Next we analyzed the centroid error in prediction error by the ten modalities. In WTR eyes, the centroid error in prediction error in both IOLMaster and SimK regarding only anterior surface power ranged from 0.50 to 0.61 D, aligned along the vertical axis (Fig. 2a). In ATR eyes, the centroid error in prediction error ranged from 0.20 to 0.39 D, aligned along with the vertical axis (Fig. 2b). With application of the Baylor nomogram and Barrett toric calculator based on IOLMaster and SimK, respectively, the centroid error in prediction error ranged from 0.24 to 0.47 D in WTR eyes and 0.12 to 0.54 D in ATR eyes, aligned all along the horizontal axis (Fig. 2a and b). With the four modalities (wavefront, TNP, TCP, and vector) combining anterior and posterior surface powers, the centroid error in prediction error ranged from 0.03 to 0.51 D. They aligned along with diverse axis in both WTR and ATR eyes (Fig. 2a and b).

As a subset analysis, the prediction errors were further divided into WTR/ATR and oblique prediction errors, respectively. They were then aggregated as a mean prediction error (Table 2). In WTR eyes, there was a significant negative mean WTR/ATR prediction error (*p* < 0.05 with Bonferroni correction) in IOLMaster, SimK, and wavefront at both 1- and 3- month indicating overestimation in corneal astigmatism but a positive mean in Baylor-IOLMatser and Baylor-SimK implying underestimation in corneal astigmatism. Meanwhile, TNP, TCP and vector in WTR eyes showed minimal WTR/ATR prediction error (range: −0.07 to 0.12 D) lesser than 0.3 D [12].

**Table 2:** Mean corneal astigmatism prediction error and intraclass correlation coefficient between estimated and measured (or calculated) corneal astigmatism

	IOLMaster		SimK		Baylor - IOLMaster		Baylor - SimK		Barrett - IOLMaster		Barrett - SimK		Wavefront		TNP		TCP		Vector	
	1 M	3 M	1 M	3 M	1 M	3 M	1 M	3 M	1 M	3 M	1 M	3 M	1 M	3 M	1 M	3 M	1 M	3 M	1 M	3 M
WTR eyes																				
Mean prediction error (D)																				
WTR/ATR	−0.33^*^	−0.42^*^	−0.46^*^	−0.56^*^	0.54^*^	0.45^*^	0.57^*^	0.48^*^	0.32^*^	0.23	0.38^*^	0.29	−0.26^*^	−0.35^*^	0.12	0.03	0.02	−0.07	0.06	−0.03
Oblique	−0.25^*^	−0.28^*^	−0.23	−0.25^*^	−0.23	−0.26	−0.18	−0.22	−0.20	−0.07	−0.18	−0.21	−0.19	−0.22	0.04	0.01	0.05	0.02	−0.16	−0.19
ICC of																				
WTR/ATR	0.74	0.66	0.92	0.87	0.75	0.67	0.90	0.83	0.81	0.78	0.83	0.82	0.94	0.90	0.70	0.61	0.69	0.61	0.94	0.90
Oblique	0.60	0.43	0.79	0.84	0.60	0.45	0.71	0.80	0.41	0.46	0.42	0.49	0.79	0.82	0.53	0.62	0.53	0.63	0.76	0.82
ATR eyes																				
Mean prediction error (D)																				
WTR/ATR	−0.34	−0.22	−0.17	−0.15	0.06	0.18	0.43^*^	0.55^*^	0.31	0.43	0.14	0.26	0.21	0.33	−0.26	−0.14	−0.17	−0.05	−0.08	0.04
Oblique	−0.20	0.02	−0.06	0.16	−0.06	0.16	−0.05	0.17	−0.18	0.04	−0.01	0.20	0.17	0.39^*^	0.06	0.28	0.07	0.29	−0.09	0.13
ICC of																				
WTR/ATR	0.41	0.32	0.71	0.56	0.21	0.16	0.52	0.47	0.41	0.32	0.47	0.36	0.65	0.75	0.67	0.56	0.59	0.47	0.74	0.74
Oblique	0.43	0.51	0.64	0.64	0.14	0.18	0.50	0.66	0.45	0.52	0.38	0.42	0.57	0.60	0.57	0.60	0.54	0.76	0.77	0.78

*TNP* true net power, *TCP* total corneal refractive power, *M* month, *WTR* with-the-rule, *ATR* against-the-rule, *D* diopters, *ICC* Intraclass correlation coefficient

^*^Significantly different from zero (all *P* < .05)

The WTR/ATR prediciton error in ATR eyes tended to underestimate corneal astigmatism in IOLMaster and SimK but to overestimate in Baylor-IOLMaster, Baylor-SimK, Barrett-IOLMaster, and Barrett-SimK even if no statistical significance in all ten modalities (*p* > 0.05) except Baylor-SimK. The vector in ATR eyes displayed the lowest WTR/ATR prediction error (range: −0.08 to 0.04 D). Overall, a trend toward overcorrection in WTR eyes, and undercorrection of corneal astigmatism in ATR eyes, was found in anterior surface-based modalities including IOLMaster and SimK but undercorrection in WTR and overcorrection in ATR in nomogram or formula-based modalities including Baylor-IOLMaster, Baylor-SimK, Barrett-IOLMaster, and Barrett-SimK. Notably the vector showed the rarefaction in WTR/ATR prediction error in both WTR and ATR eyes. No significant oblique prediction error was found in the majority of modalities except for IOLMaster and SimK of WTR eyes and wavefront of ATR eyes.

### ICC between estimated and measured (or calcualted) corneal astigmatism

Results of ICC of measured (or calcualted) astigmatisms are shown in Table 2. In WTR eyes, excellent agreement index (WTR/ATR ICC > 0.8) [11] was observed in WTR/ATR with SimK (ICC: 0.92 and 0.87 at 1- and 3- month, respectively), Baylor-SimK (0.90 and 0.83, respectively), Barrett-SimK (0.83 and 0.82, respectively), wavefront (0.94 and 0.90, respectively), and vector (0.94 and 0.90, respectively). In terms of oblique ICC, substantial agreement index (ICC > 0.6) [11] was noted with SimK (0.79 and 0.84, respectively), Baylor-SimK (0.71 and 0.80, respectively), wavefront (0.79 and 0.82, respectively), and vector (0.76 and 0.82, respectively). In ATR eyes, substantial agreement was observed in WTR/ATR ICC with wavefront (0.65 and 0.75, respectively) and vector (0.74 and 0.74, respectively). Regarding the oblique ICC, only vector (0.77 and 0.78, respectively) showed ICC higher than 0.6.

## Discussion

It is well known that the utilization of conventional keratometric device regarding only anterior corneal surface can result in a significant residual astigmatism error for the determination of toric IOL cylinder. Overcorrection can occur in WTR astigmatism, while undercorrection can occur in ATR astigmatism [4, 13, 14]. In this study, negative value (WTR eyes; overcorrection, ATR eyes; undercorrection) of WTR/ATR prediction error was also found in conventional keratometry, IOLMaster. This error could be mainly attributed to a concealed posterior astigmatism mostly aligning along the vertical steep axis which cannot be measured in conventional keratometry [4, 13]. To overcome this pitfalls, the Baylor nomogram and Barrett toric calculator were introduced to adjust toric IOL power to account for posterior corneal astigmatism by regression analysis and theoretical model, respectively [4, 5]. To measure the actual corneal astigmatism, Pentacam and Galilei are the alternative devices using Schiempflug imaging that takes posterior corneal surface into account [8, 15]. However, a measurement of true corneal astigmatism for toric IOL selection is still controversial since the Baylor nomogram and Barrett toric calculator do not reflect the actual posterior astigmatism, and the usefulness of Schiempflug imaging devices is not universally validated for toric IOL implantation. To solve the current issues for toric IOL implantation, we investigated and compared the accuracy of astigmatism measurements derived from diverse modalties, including anterior surface-based modality (IOLMaster and SimK), adjusted modality (Baylor-IOLMaster, Baylor-SimK, Barrett-IOLMaster, and Barrett-SimK), and both surface-based modality (wavefront, TNP, TCP, and vector).

Similar to previous clinical outcomes in Pentacam measurements for toric IOL implantation [5, 16], wavefront, TNP, TCP, and vector were superior to IOLMaster and SimK in terms of prediction error or ICC. Among them, vector was the best way to predict corneal astigmatism for toric IOL selection by showing minimum prediction error with substantial agreement with the estimated preoperative corneal astigmatism values for both WTR and ATR eyes.

With a careful subgroup analysis in prediction error, significant WTR/ATR prediction errors were found in the IOLMaster, SimK, Baylor-IOLMaster, Barrett-IOLMaster of WTR eyes and Baylor-SimK of ATR eyes at two time periods (postoperative 1- and 3-month) but no significant error in the majority of both surface-based modalities (except wavefront in WTR eyes). Notably, a rare prediction error of vector was observed in both WTR and ATR eyes. In contrast, Koch et al. [4] have reported significant negative values of WTR/ATR prediction errors (overcorrection in WTR eyes and undercorrection in ATR eyes) in all keratometries or topographic devices except an adequate correction in ATR astigmatism (but not in WTR astigmatism) using the Galilei Placido-dual Schiempflug analyzer [4]. The following two possible reasons might explain such difference in astigmatism correction between the two studies. First, the adjustment of SICA for the estimation of preoperative corneal astigmatism used in the current study might have influenced the non-significant difference to the zero diopter of the WTR/ATR prediction error. This was not performed in the other study [4]. Second, the disparity of correction in WTR astigmatism between Pentacam and Galilei might have attributed to the measurement difference between the two devices. In the study of Koch’s et al. [4], the prediction errors of WTR eyes between anterior keratometry (−0.47 to −0.60 D) and Galillei (−0.57 D) were similar to each other. Therefore, underestimation of posterior K value in Gailei cannot be rejected. However, in the current study, different ranges of the mean WTR/ATR prediction errors between IOLMaster (−0.33 to −0.42 D), SimK (−0.46 to −0.56 D), and vector (−0.03 to 0.06 D) were found, probably reflecting the influence of mean posterior corneal astigmatism (0.56 D) on vector of WTR eyes.

To further analyze the accuracy of Pentacam measurements for toric IOL implantation, we accessed their ICC with estimated corneal astigmatism. ICC is a useful tool in assessing both consistency and agreement in evaluation of measurement error [17]. In this study, we found excellent agreement (WTR/ATR ICC > 0.8) for SimK, Baylor-SimK, Barrett-SimK, wavefront, and vector on WTR eyes and substantial agreement (WTR/ATR ICC > 0.6) for wavefront and vector on ATR eyes. Among the ten modalities, only vector showed lesser mean prediction error than the minimum allowed astigmatism value (0.3 D) [12] simultaneously with substantial agreement index (both WTR/ATR and oblique ICC > 0.6) on both WTR and ATR eyes. Although the mean WTR/ATR prediction errors in both TNP and TCP were close to zero diopter, the WTR/ATR ICCs of them (0.61 ~ 0.70 in WTR eyes, 0.47 ~ 0.67 in ATR eyes) were significantly lower than those of vector (0.90 ~ 0.94 in WTR eyes, 0.74 in ATR eyes), implying the superiority of vector for predicting preoperative corneal astigmatism.

Intriguingly, rather lower values of vector in both WTR/ATR and oblique ICC for ATR eyes than that for WTR eyes was found, indicating the lesser reliability of vector for toric selection of ATR eyes. This complicated result is supported by a previous study showing that ATR eyes have larger estimation errors in astigmatism magnitude than WTR eyes [18]. The greater amount of SICA in ATR eyes than that in WTR eyes and the potential measurement error of posterior K from Pentacam, especially in ATR eyes, might have contributed to the lower ICC of vector in ATR eyes. It has been acknowledged that the greater SICA is associated with older age [19], and lower corneal hysteresis and resistance factor [20]. The discrepancy between the assumed and substantial SICA appears to be inevitable. Larger samples are needed to prove these possibilities.

It has been suggested that the centroid errors in prediction error when using Pentacam measurements are lower for toric IOL selection compared to conventional keratometry measurements [5, 16]. Similarly, this study revealed that there was minimal WTR/ATR prediction error when using TNP, TCP, and vector derived from Pentacam. On the other hand, the WTR/ATR ICCs for both TNP (0.56 ~ 0.70) and TCP (0.47 ~ 0.69) were significantly lower than that of vector (0.74 ~ 0.94). TNP is calculated by adding sagittal curvature values of the anterior and posterior corneal planes while TCP reflects both the exact light path and the corneal surface curvature [16]. Both TNP and TCP do not appear to be appropriate for the determination of true corneal astigmatism. The reason why TNP and TCP were unpredictable for toric IOL selection in this study remains unclear. However, a previous study has reported the concern about the accuracy of TNP for predicting postoperative astigmatism after cataract surgery [21]. The usefulness of TNP and TCP for toric IOL implantation should be further validated.

Interestingly, the application of Baylor nomogram and Barrett formula in our study yielded WTR residual refractive astigmatism (undercorrection in WTR eyes and overcorrection in ATR eyes), which is opposite to the outcome of ATR residual refractive astigmatism when using the anterior surface-based modalities. A similar result was revealed in a previous study comparing anterior keratometry (OLCR device) and Baylor nomogram [16]. The Baylor nomogram and Barrett toric calculation provide an adjusted corneal reflecting a predicted posterior astigmatism based on anterior keratometry, ACD, or axial length [4, 7]. Although leaving WTR refractive astigmatism would be helpful due to the anticipated ATR shift in most eyes [4], the adjustment of posterior corneal astigmatism based on regression analysis (Baylor nomogram) or theoretical formula (Barrett toric calculation) appears to be overcompensated caused by a methodologic error itself.

Despite our vigorous effort to decrease the error in estimating preoperative corneal astigmatism, there are some pitfalls to predict the estimated preoperative corneal astigmatism. IOL tilt might have contributed to rest prediction error [22]. Although the significant IOL tilt was not found from this study, the possibility of subtle IOL tilt could be associated with the remnant prediction error [23].

ACD and effective lens position (ELP) were not considered when selecting the IOL toricity in our study. Precise preoperative ACD measurement ACD or ELP estimation is needed to to predict IOL corneal plane cylinder power [24]. Eom has announced the influence of ELP with adjustment of AcrySof toric cylinder power up to 0.2 D [25]. Further study is required to involve the effect of ACD or ELP.

## Conclusion

In conclusion, the outcomes of toric IOL implantation could be improved by adjusting posterior corneal surface astigmatism using Schiempflug imaging device Pentacam. Specifically, the vector, a vector summation modality using both anterior and posterior corneal surfaces measured from Pentacam was feasible for predicting the appropriate preoperative corneal astigmatism. It could be used as a promising tool for determining toric IOL cylinder power.

## Abbreviations

ACD: Anterior chamber depth
ATR: Against-the-rule
ELP: Effective lens position
ICC: Intraclass correlation coefficient
IOL: Intraocular lens
K: Keratometry
SICA: Surgical induced corneal astigmatism
WTR: With-the-rule
